# Archeological neuroimmunology: resurrection of a pathogenic immune response from a historical case sheds light on human autoimmune encephalomyelitis and multiple sclerosis

**DOI:** 10.1007/s00401-020-02239-2

**Published:** 2020-10-29

**Authors:** Eduardo Beltrán, Manuela Paunovic, David Gebert, Emine Cesur, Markus Jeitler, Romana Höftberger, Joachim Malotka, Simone Mader, Naoto Kawakami, Edgar Meinl, Monika Bradl, Klaus Dornmair, Hans Lassmann

**Affiliations:** 1 Institute of Clinical Neuroimmunology, University Hospital and Biomedical Center, Ludwig-Maximilians University Munich, Munich, Germany; 2 Department of Neuroimmunology, Center for Brain Research, Medical University of Vienna, Spitalgasse 4, 1090 Vienna, Austria; 3 Core Facility Genomics, Medical University Vienna, Vienna, Austria; 4 Division of Neuropathology and Neurochemistry, Department of Neurology, Medical University of Vienna, Vienna, Austria; 5 Munich Cluster for Systems Neurology (SyNergy), Munich, Germany

**Keywords:** Next generation sequencing, Myelin oligodendrocytes glycoprotein, Multiple sclerosis (MS), Human autoimmune encephalitis

## Abstract

Aim of our study was to identify the target auto-antigen in the central nervous system recognized by the immune system of a unique patient, who died more than 60 years ago from a disease with pathological changes closely resembling multiple sclerosis (MS), following a misguided immunization with lyophilized calf brain tissue. Total mRNA was isolated from formaldehyde fixed and paraffin embedded archival brain tissue containing chronic active inflammatory demyelinating lesions with inflammatory infiltrates rich in B-lymphocytes and plasma cells. Analysis of the transcriptome by next generation sequencing and reconstruction of the dominant antibody by bioinformatic tools revealed the presence of one strongly expanded B-cell clone, producing an autoantibody against a conformational epitope of myelin oligodendrocytes glycoprotein (MOG), similar to that recognized by the well characterized monoclonal anti-MOG antibody 8-18C5. The reconstructed antibody induced demyelination after systemic or intrathecal injection into animals with T-cell mediated encephalomyelitis. Our study suggests that immunization with bovine brain tissue in humans may—in a small subset of patients—induce a disease with an intermediate clinical and pathological presentation between MS and MOG-antibody associated inflammatory demyelinating disease (MOGAD).

**Electronic supplementary material:**

The online version of this article (10.1007/s00401-020-02239-2) contains supplementary material, which is available to authorized users.

## Introduction

Multiple sclerosis is seen as an autoimmune disease, although so far no MS-specific pathogenic autoimmune reaction has been identified [15, 16]. Despite this caveat, experimental autoimmune encephalomyelitis (EAE) in various different animal species serves as a key disease model for MS [11, 25]. Long before the first accounts of EAE in experimental animals [39, 44] it was already known from experience with rabies vaccination that active sensitization of humans with brain tissue can trigger a demyelinating encephalomyelitis [2, 21]. Formal proof for the autoimmune nature of this condition was provided by observations, showing that such conditions also followed direct immunization with brain tissue [23, 40]. Epidemiological studies on rabies vaccination associated autoimmune encephalomyelitis, based on more than 300.000 individuals, described an incidence of this neurological complication of 1:1000. The neurological manifestation in the vast majority of patients consisted of acute inflammatory polyneuropathy and acute disseminated encephalomyelitis [1, 45]. However, a small subset of patients developed an inflammatory demyelinating disease closely similar to that seen in multiple sclerosis patients [13, 19, 40, 48]. Knowledge of the nature of the human autoimmune encephalitis (hAE) response in such cases may lead to fundamental new insights into the pathogenesis of human inflammatory demyelinating diseases, including MS.

We have addressed this question in our present study by resurrecting the antibody response from the archival formaldehyde fixed and paraffin embedded (FFPE) brain tissue of the patient described by Jellinger and Seitelberger [19]. Our studies showed that the formation of the MS-like inflammatory demyelinating lesions in this case was associated with one strongly expanded B-cell clone producing an auto-antibody, termed Ab-hAE. Thus, we analyzed the transcriptome of the FFPE tissue by next-generation sequencing, reconstructed the dominant antibody by bioinformatic tools, and expressed it recombinantly yielding recombinant Ab-hAE (rAb-hAE). This antibody recognizes a conformational epitope of myelin oligodendrocyte glycoprotein (MOG) that is structurally similar to the epitope of the pathogenic antibody 8-18C5 [7, 26, 27]. The reconstructed recombinant antibody induced demyelination in experimental animals with inflammatory brain disease.

## Materials and methods

### The hAE-patient

The FFPE brain blocks used in this study are from a 51-year-old male patient who died 1958. This patient suffered from a mild slowly progressive hemi-Parkinson syndrome, which started 4 years before his death, and for which he was treated over 17 months with multiple injections of lyophilized calf brain and placenta cells. After the last injection the patient developed progressive right-sided hemiparesis followed by a rapid onset of neurological disease and died 7 weeks later due to cardiorespiratory failure [19]. Detailed neuropathological analysis revealed a pathological phenotype fulfilling all criteria of Marburg’s type of acute MS [13, 31]. The current study was approved by the Ethic Commission of the Medical University Vienna and performed with the license of the Austrian Ministry for Science and Research (535/2004).

### RNA isolation

For RNA isolation from FFPE embedded brain tissue, the commercial High Pure FFPE RNA Micro Kit (Roche, Vienna, Austria) was used with the modification that after deparaffination, treatment with citraconic anhydrate (CCA) [33] was added. Briefly, one-to-two tissue sections (each 8 µm thick) were deparaffinized in xylene (VWR, Vienna, Austria), rehydrated and dried at 55 °C for 15 min. Afterwards, an incubation with 1% CCA (VWR; diluted in absolute ethanol, adjusted to pH 7.40 with NaOH, resulting in a final ethanol concentration of approx. 60%) at 98 °C for 45 min at 1200 rpm in a ThermoMixer C (Eppendorf, Vienna, Austria) was performed and followed by a washing step with 70% ethanol. The tissue was lysed for 10 h at 55 °C in lysis buffer (tissue lysis buffer with proteinase K and 10% sodium dodecyl sulfate (SDS) followed by an incubation at 70 °C for 20 min to inactivate proteinase K. The remaining RNA isolation steps were done according to the manufacturers’ instructions. The RNA was finally eluted in 20 µl elution buffer and stored at − 80 °C. The RNA quality was determined by Agilent 2100 Bioanalyzer using Agilent RNA Pico Chips (Agilent, Cambridge, UK). As for FFPE tissues the 18S and 28S rRNA peaks are not identifiable, we used the DV200 value, which gives the percentage of RNA-fragments with a length longer than 200 nucleotides, as quality criterion. Samples with a DV200 of approximately 50% were classified as suitable for further processing.

### Reverse transcription, NGS library preparation, and sequencing

3 µl of RNA were mixed with 1 µl of a reverse-transcription primer pool (10 µM, list of primers see Table 1). The mix was incubated at 72 °C for 3 min to denature RNA and RT primers, followed by an incubation at 42 °C for 2 min. First-strand cDNA was synthesized using the SmartScribe kit (Clontech) in conjunction with a template switching oligo. RT reaction was performed at 42 °C for 60 min, followed by a final step at 70 °C for 10 min. To clean the RT reaction product Agencourt XP beads (Beckman Coulter) were used in a 1:1.8 v/v ratio according to the manufacturers’ instructions. Then 3 µl of the cleaned RT product were mixed with 12.5 µl Phusion 2X Master Mix (New England Biolabs), 0.1 µl TSO-PCR primer (100 µM, see Table 2), 0.1 µl BCR specific primer pool (100 µM, see Table 2) and 9.3 µl RNAse-free water. First PCR amplification was performed for 25 cycles: 98 °C for 20 sec, 60 °C for 20 sec and 70 °C for 50 sec, followed by a final extension at 70 °C for 10 min. A 1X bead clean-up was performed afterwards. A second PCR amplification to further enrich the target Ig fragments and introduce Nextera adapters was performed with the following components: 1 µl of cleaned PCR product, 12.5 µl Phusion 2X Master Mix, 0.1 µl Nextera-TSO-primer pool (100 µM, sequences see Table 3), 0.1 µl Nextera-BCR-primer pool (100 µM, sequences see Table 3) and 11.3 µl RNase-free water. The same PCR conditions as described for the first PCR were used. The PCR product was subjected to a 0.6–1× double sided size selection clean-up, diluted to a final concentration of 1 ng/µl, and indexed using the Nextera XT Index Kit v2 (Illumina). Indexing PCR was performed for 12 cycles: 98 °C for 10 s, 55 °C for 30 s and 72 °C for 30 s; followed by a final extension at 72 °C for 5 min (Ig-seq). The KAPA Stranded RNA Sequencing Kit with RiboErase HMR (Roche, Vienna, Austria) was used according to the manufacturers’ instructions to generate libraries for whole transcriptome analysis (RNAseq). The obtained Ig-seq and RNA-seq libraries were subjected to quality control using an Agilent Bioanalyzer and sequenced at the Core Facility Genomics, Medical University of Vienna. Paired-end read (150-bp) sequencing was performed on a NextSeq 500 sequencer (Illumina).

**Table 1:** Primer sequences for step 1 in generation of antibody-repertoire specific libraries

Primer pool name	Primer name	Sequence (5′–3′)
Reverse-transcription primer pool (10 µM)	HG_RT-in	AGAGGTGCTCTTGGAG
	Cmu_RT-in	GGGAATTCTCACAGGAG
	Kappa_RT-in	CACACAACAGAGGCAG
	Lambda_RT-in	CACCAGTGTGGCCTTG
Template switch oligo (10 µM)	TSO	/5Biosg/AAGCAGTGGTATCAACGCAGAGTACATrGrG+G

**Table 2:** Primer sequences for step 2 in generation of antibody-repertoire specific libraries

Primer pool name	Primer name	Sequence (5′–3′)
TSO-PCR primer (100 µM)	TSO-PCR primer	AAGCAGTGGTATCAACGCAG
BCR specific primer pool (100 µM)	unb_HG_IN new	AGAGGTGCTCTTGGAG
	unb_Cmu_OUT	GGGAATTCTCACAGGAG
	unb_Kappa_OUT	CACACAACAGAGGCAGTTCC
	unb_Lambda_OUT	CACCAGTGTGGCCTTGTTGG

**Table 3:** Primer sequences for step 3 in generation of antibody-repertoire specific libraries

Primer pool name	Primer name	Sequence (5′–3′)
Nextera-TSO-primer pool (100 µM)	Nextera_NN_TSO-2ndPCR	TCGTCGGCAGCGTCAGATGTGTATAAGAGACAG**NN**CAGTGGTATCAACGCAGAG
	Nextera_NNN_TSO-2ndPCR	TCGTCGGCAGCGTCAGATGTGTATAAGAGACAG**NNN**CAGTGGTATCAACGCAGAG
	Nextera_NNNN_TSO-2ndPCR	TCGTCGGCAGCGTCAGATGTGTATAAGAGACAG**NNNN**CAGTGGTATCAACGCAGAG
Nextera-BCR-primer pool (100 µM)	Nextera_NN_ IGHJ	GTCTCGTGGGCTCGGAGATGTGTATAAGAGACAG**NN**GAGGAGACGGTGACCRKGGT
	Nextera_NNN_ IGHJ	GTCTCGTGGGCTCGGAGATGTGTATAAGAGACAG**NNN**GAGGAGACGGTGACCRKGGT
	Nextera_NNNN_ IGHJ	GTCTCGTGGGCTCGGAGATGTGTATAAGAGACAG**NNNN**GAGGAGACGGTGACCRKGGT
	Nextera_NN_Kappa	GTCTCGTGGGCTCGGAGATGTGTATAAGAGACAG**NN**GAAGATGAAGACAGATGGTGC
	Nextera_NNN_Kappa	GTCTCGTGGGCTCGGAGATGTGTATAAGAGACAG**NNN**GAAGATGAAGACAGATGGTGC
	Nextera_NNNN_Kappa	GTCTCGTGGGCTCGGAGATGTGTATAAGAGACAG**NNNN**GAAGATGAAGACAGATGGTGC
	Nextera_NN_Lambda	GTCTCGTGGGCTCGGAGATGTGTATAAGAGACAG**NN**GCTTGGAGCTCCTCAGAGG
	Nextera_NNN_Lambda	GTCTCGTGGGCTCGGAGATGTGTATAAGAGACAG**NNN**GCTTGGAGCTCCTCAGAGG
	Nextera_NNNN_Lambda	GTCTCGTGGGCTCGGAGATGTGTATAAGAGACAG**NNNN**GCTTGGAGCTCCTCAGAGG

### NGS analysis

Removal of adapters and quality trimming was performed and only trimmed sequences were used for downstream analysis. Sequences from the Ig-seq libraries were aligned to Ig Heavy, Kappa- and Lambda-chain genes. The sorted reads were subjected to MiGEC-CdrBlast [41] for extraction of Ig chains, leading to the identification of CDR3 sequences of all Ig chains in the data. Plots to visualize the repertoire clonality were generated with VDJtools [42]. Sequences from the RNA-seq libraries were blasted against the human Ig loci using IgBLAST from the National Center for Biotechnology Information. Only reads with at least 80% alignment to VDJ genes were used to identify V, D, and J gene segments. Reads with the YYC motif were used to search for CDR3 sequences identified on the Ig-seq analysis. Then these reads where used as a template, while all the other mapped reads where used to start reconstructing the full-length Ig heavy- and light-chains (H- and L-chains) from the 3′- to the 5′-end of the V segment by overlapping reads.

### Cloning, expression and purification of recombinant immunoglobulins

The variable regions of the H- and L-chain of antibody Ab-hAE extended for the restriction sites SacII and SalI (H-Chain) and KasI and BssHII (L-Chain) were synthetized by Geneart (Regensburg, Germany). Both chains were each cloned into the expression plasmid pTT5 that contained the conserved regions of the human IgG1 H- and kappa chains, co-transfected into HEK293-Expi cells (Thermo Fisher), purified by immobilized metal affinity chromatography, and characterized as described for the control antibodies rOCB-MS3-s1, rOCB-NB1-s13, and r8-18C5 [5]. We use the prefix “r” to indicate recombinant antibodies. Throughout this manuscript, 8-18C5 refers to the original murine monoclonal MOG-specific antibody [27], while r8-18C5 refers to its recombinant derivative containing the constant regions of human IgG1 [5]. Analogously, Ab-hAE refers to the antibody in the patient, while rAb-hAE refers to its recombinant derivative.

### Immunohistochemistry

#### Basic characterization of B-cell lineage cells in the hAE case

2-5 µm thick tissue sections were deparaffinized, rehydrated, and the endogenous peroxidase was blocked with 0.2% H_2_O_2_ in methanol for 30 min. Depending on the antibodies used, antigen retrieval was performed for 1 h by steaming of the tissue sections in 1 mM EDTA in 10 mM Tris buffer (pH 9.0), or for 15 min at 37 °C in 0.03% protease (Type XXIV; Sigma, Vienna, Austria) in 1 × phosphate-buffered saline (PBS). Afterwards, the sections were rinsed in 0.1 M PBS or Tris-buffered saline (TBS) and incubated with 10% fetal calf serum (FCS, LifeTech Austria, Vienna, Austria) in 1 × DAKO Wash Buffer for 20 min at room temperature to reduce non-specific background, and incubated overnight at 4 °C with primary antibodies (diluted with 10% FCS in 1 × DAKO Wash Buffer). Then, the sections were washed 3 × in TBS, and incubated for 1 h at room temperature (RT) with biotinylated secondary antibodies (diluted with 10% FCS in 1 × DAKO Wash Buffer). After a further washing step (3 × TBS), peroxidase-conjugated streptavidin (Jackson Immuno Research, Cambridge, UK, diluted 1:100 with 10% FCS in 1 × DAKO Wash Buffer) was applied for 1 h at room temperature (RT). For some antibodies, staining was enhanced by biotinylated tyramine amplification (catalyzed signal amplification = CSA [3]. Afterwards, the sections were washed with 1 × PBS and incubated for 30 min with peroxidase-conjugated streptavidin (diluted as described before). The staining reactions were finalized by adding 3,3ʹ-diaminobenzidine tetrahydrochloride hydrate (DAB; ≥ 97.5%; Sigma, Vienna, Austria, containing 0.01% hydrogen peroxide). Before mounting the sections with Eukitt © (Sigma), the sections were counterstained with hematoxylin (VWR, Vienna, Austria). All antibodies and the respective staining conditions are summarized in Table 4.

**Table 4:** Antibodies used for staining of human tissue

Antibody	Source	Target	Antibody dilution	Pre-treatment	Secondary antibody (Jackson Immuno Research, Cambridge, UK)	Tyramine amplification
CD79a	Agilent, Cambrigde, UK	B-cell antigen receptor complex-associated protein alpha chain	1:100	EDTA 9.0	Biotin-α-Mouse (1:500)	–
CD19	Eubio, Vienna, Austria	C-terminal cytoplasmic tail sequence of human CD19, expressed on B cells	1:500	EDTA 9.0	Biotin-α-Mouse (1:500)	CSA (1:1000)
CD20	Thermo Scientific, Vienna, Austria	Non-immunoglobulin differentiation antigen CD20 of B cells	1:100	EDTA 9.0	Biotin-α-Mouse (1:500)	–
CD27	Biotechne, Abingdon, UK	Human CD27/TNFRSF7 expressed on memory B cells	1:1000	EDTA 9.0	Biotin-α-Rabbit (1:1000)	CSA (1:1000)
CD38	Eubio, Vienna, Austria	C-Terminus of human CD38 expressed on plasmablasts	1:1000	EDTA 9.0	Biotin-α-Rabbit (1:1000)	–
CD138	BioRad AbD Serotech, Puchheim, Germany	Syndecan-1 (CD138) expressed on all plasma cells	1:250	EDTA 9.0	Biotin-α-Mouse (1:500)	–
Ig (Biotin-α-Human)	Szabo Scandic, Vienna, Austria	Human IgG and light chains of other human immunoglobulins	1:1000	Protease	–	–

#### Testing of antibody binding to human, primate and rat brain sections

Commercial tissue sections from cerebellum from primates (FB 1111-1010-17, Euroimmun) were stained with rAb-hAE, r8-18C5, and rOCB-NB1-s13 (used as negative control [5]) at 20 µg/ml in 20 mM sodium phosphate buffer, pH 7.4, 150 mM NaCl, 0.02% Tween 20 (PBST) for 30 min at room temperature. After three washing steps in PBST, mouse anti-human IgG1 Hinge-BIOT (Southern Biotech) was added at a 1:100 dilution in PBST and incubated for 30 min at room temperature. After two washing steps, Streptavidin Alexa FluorTM488 conjugate (Thermo Fisher, 1:400) was added for 30 min. Slides were washed twice in PBST and then incubated for 5 min in PBST containing DAPI solution (1:12.500, Thermos Scientific). The slides were then mounted in embedding medium. Images were taken with a Leica DM IL LED Fluo microscope with a DFC3000G camera. Additional immunohistochemistry was performed on brain sections from a control patient without neurological disease (female, age 71), from the huAE index patient, and from Lewis rats, using rAb-hAE, r8-18C5 (positive control), and rAb-OCB-MS3-s1 (negative control) in a dilution of 1:1000. Antibody binding was visualized by the biotin avidin technique, described above.

#### Characterization of the demyelinating activity of rAb-hAE

Immunohistochemistry was essentially performed as described above, using antibody and staining conditions as summarized in Table 5. For the W3/13 antibody, antigen retrieval was performed for 1 h by steaming of the tissue sections in 10 mM citrate buffer (pH 6.0).

**Table 5:** Antibodies used for staining of rodent tissue

Antibody	Source	Target	Antibody dilution	Pre-treatment	Secondary antibody (Jackson Immuno Research, Cambridge, UK)	Tyramine amplification
CNP	Sternberger Monoclonals Incorporated, Maryland, USA	2′,3′-Cyclic-nucleotide 3′-phosphodiesterase (CNPase) present in oligodendrocytes and myelin	1:2000	EDTA 8.5	Biotin-α-Mouse (1:500)	–
ED1	VWR, Vienna, Austria	Rat CD68 protein expressed by macrophages and activated microglia	1:10,000	EDTA 8.5	Biotin-α-Mouse (1:500)	–
C9neo	Details see [36]	activated complement component C9	1:2000	Protease	Biotin-α-Rabbit (1:1000)	–
CD3	Agilent, Cambridge, UK	CD3 on T cells	1:1000	EDTA 8.5	Biotin-α-Rabbit (1:1000)	–
W3/13	Abcam, Cambridge, UK	CD43 glycoprotein on T cells and neutrophils	1:50	Citrat	Biotin-α-Mouse (1:500)	–
Ig (Biotin-α-Human)	Szabo Scandic, Vienna, Austria	Human IgG and light chains of other human immunoglobulins	1:1000	Protease	–	–

### Flow cytometry

Recognition of wild-type human, rat, and mouse MOG (hMOG, rMOG, and mMOG, respectively) and of six hMOG mutants with site-specific amino acid substitutions (N31D, R9G/H10Y, P42S, R86Q, S104E and H103A/S104E; [32]) by rAb-hAE, r8-18C5, and the control antibody rOCB-MS3-s1 was analyzed by flow cytometry. To this end, COS-7 cells were transiently transfected with cDNA of the above MOG species variants cloned into plasmids pEGFP-N1 or pcDNA 6.2C-EmGFP-GW-TOPO [32] leading to the expression of enhanced GFP (EGFP)- or emerald GFP- (EmGFP)-tagged fusion proteins using the SE Cell Line 4D-Nucleofector X Kit L (LONZA, Basel, Switzerland) according to the manufacturer's protocol.

Cells were incubated in fetal calf serum-supplemented RPMI 1640 media (Thermo Fisher) for 48 h, detached by 0.05% trypsin–EDTA (Thermo Fischer), washed three times, and resuspended in FACS-buffer (1% FCS in 1 × PBS, Thermo Fischer). Cells were incubated with 0.033–100 µg/ml of either antibody for 30 min on ice, washed three times, and incubated with the goat anti-human IgG (1:500 diluted, biotin-SP conjugated, Jackson Immunoresearch) for 30 min on ice. After three washing steps, they were stained with streptavidin-Alexa-Fluor647 (1:2000, Jackson Immunoresearch) for 30 min on ice. Finally, cells were washed and suspended in FACS-buffer containing propidium iodide (1:6000) and analyzed using a BD FACSVerse™ (BD Biosciences) and the software FlowJo™ (Tree Star). Only EGFP-positive, i.e., efficiently transfected cells were used of analysis.

### Animals

7–8-week-old Lewis female rats were obtained from Charles River Wiga (Sulzfeld, Germany), and were housed in the Decentral Facilities of the Institute for Biomedical Research (Medical University Vienna) or in the Core Facility Animal Models of the Biomedical Center (Ludwig-Maximilians-University Munich) under standardized conditions. All applicable international, national, and/or institutional guidelines for the care and use of animals were followed. All procedures performed in studies involving animals were in accordance with the ethical standards of the institution at which the studies were conducted. The experiments in Vienna (intraperitoneal antibody application in the course of experimental autoimmune encephalitis (EAE)) were approved by the Ethic Commission of the Medical University Vienna and performed with the license of the Austrian Ministry for Science and Research (BMBWF-66.009/0279-V/3b/2019), the experiments in Munich (intrathecal antibody injection in the course of EAE) were approved by the Government of Upper Bavaria (55.2-1-54-2532-27-2016).

### Intraperitoneal antibody application in the course of EAE

For EAE induction, activated myelin basic protein (MBP)-specific T cells (0.5 × 10^5^ cells/ml; 2 ml cell suspension per animal) were injected intraperitoneally (i.p.) on day 0, followed by an i.p. injection with the recombinant antibodies rAb-hAE (4 mg), rOCB-MS3-s1 (1 or 4 mg, negative control), or r8.18-C5 (1 mg, positive control) on day 4. As the antibody r8-18C5 has a high affinity to human and rodent myelin oligodendrocyte glycoprotein (MOG), it was used in lower concentrations than rAb-hAE and rOCB-MS3-s1. The clinical course of EAE was assessed according to the following score: 0 = healthy; 0.5 = partial loss of tail tonus; 1 = complete loss of tail tonus; 2 = unsteady gait, hind limb weakness; 3 = bilateral hind limb paralysis. 48 h after antibody injections, the animals were sacrificed with CO_2_, perfused with 4% paraformaldehyde (PFA) in phosphate-buffered saline (PBS), and dissected. The tissues (brain and spinal cord) were fixed in 4% PFA/PBS overnight at 4 °C and embedded in paraffin for histological analysis.

### Intrathecal antibody injection in the course of EAE

Freshly activated MBP-specific T cells (2 × 10^6^ cells/ml, 0.5 ml cell suspension per animal) were intravenously injected on day 0. On day 2, the animals were anesthetized by injecting 5 μg/kg Fentanyl, 2 mg/kg Midazolam, and 150 μg/kg Medetomidin. Then, OCB-MS3-s1 (100 µg or 350 µg, negative control), r8.18-C5 (100 µg, positive control) or rAb-hAE (100 µg or 350 µg) were injected into the cisterna magna in a volume of 100 ml and an injection speed of 10 μl/min. After intrathecal injection, 0.12 mg/kg Naloxon, 0.2 mg/kg Flumazenil, 0.75 mg/kg Antipamezol was injected i.p. 72 h after antibody injections, the animals were sacrificed with CO_2_ and their tissues processed for histological analysis as described above.

## Results

### Histological characterisation of lymphocytes and identification of plasma cells in the inflammatory demyelinating lesions

The pathology and immunopathology of the case of hAE has been described and illustrated in detail recently [13]. It shows many similarities with MS [24], such as a lymphocyte and macrophage dominated inflammatory reaction, large confluent periventricular demyelinated lesions with extensions of the lesions around inflamed veins (the so-called Dawson Fingers [9]), primary demyelination with partial axonal preservation [13] and reactive astrocytic scaring [13]. All lesions contained an inactive lesion core and were surrounded by a rim of active demyelination, characterized by the presence of densely packed macrophages with early myelin degradation at the expanding lesion edge [13]. Inflammatory infiltrates were dominated by CD20^+^ B-cells and CD8^+^ T-cells, while—in striking contrast to EAE in rodents—only exceptionally CD4^+^ T-cells were found. In particular, the inflammatory infiltrates revealed features of lymph follicle like inflammatory infiltrates, with segregated T-cell, B-cell and plasma cell areas [13], as they have been described in the meninges and periventricular Virchow Robins spaces in multiple sclerosis [29, 30]. Active lesion areas showed the deposition of immunoglobulin and activated complement (C9neo) at sites of active demyelination [13].

**Fig. 1:**
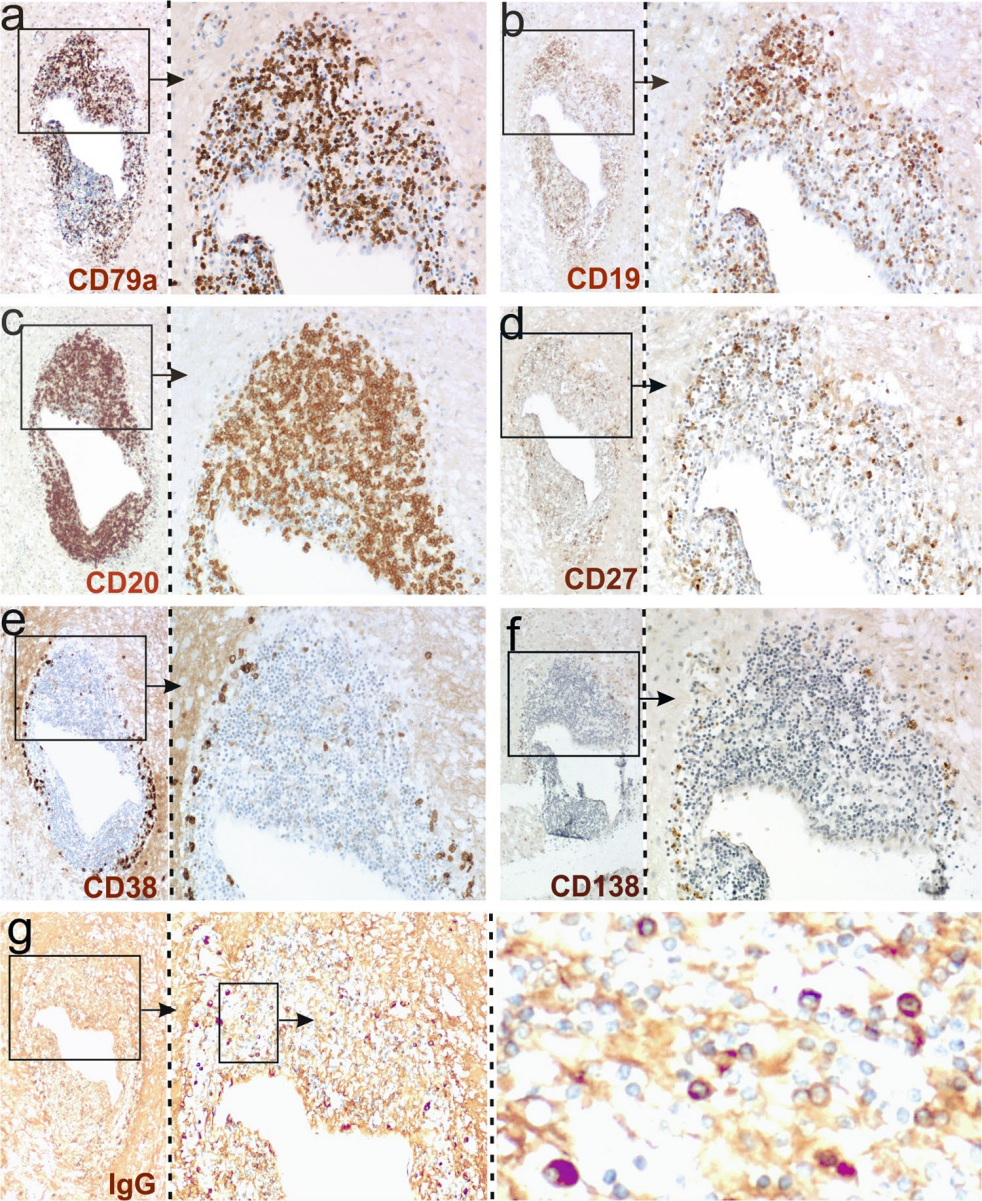
B cells and plasma cells in the brain of patient hAE. Consecutive brain sections were reacted with antibodies against CD79a (**a**), CD19 (**b**), CD20 (**c**), CD27 (**d**), CD38 (**e**), CD138 (**f**) and human IgG (**g**). Positive reaction products are brown, and the tissue was counterstained with hematoxylin to show the nuclei in blue. The boxes with arrows mark details of a large perivascular cuff which are enlarged in the adjacent pictures. Note the presence of large numbers of IgG^+^ plasma cells in (**g**)

Here we studied the B-lineage cell infiltrates in more detail. We found that the majority of the cells were CD79a^+^ CD20^+^ B-cells, Fig. 1a, c), which only in part also expressed CD19 and the activation marker CD27 (Fig. 1b, d). 10% of the lymphoid cells within the lesions were CD38^+^ plasma blasts (Fig. 1e) or CD138^+^ plasma cells (Fig. 1f), which to more than 90% contained IgG (Fig. 1g), but in a low incidence also IgM and IgA [13].

### Immunoglobulin repertoire analysis reveals a strongly expanded B cell clone

**Fig. 2:**
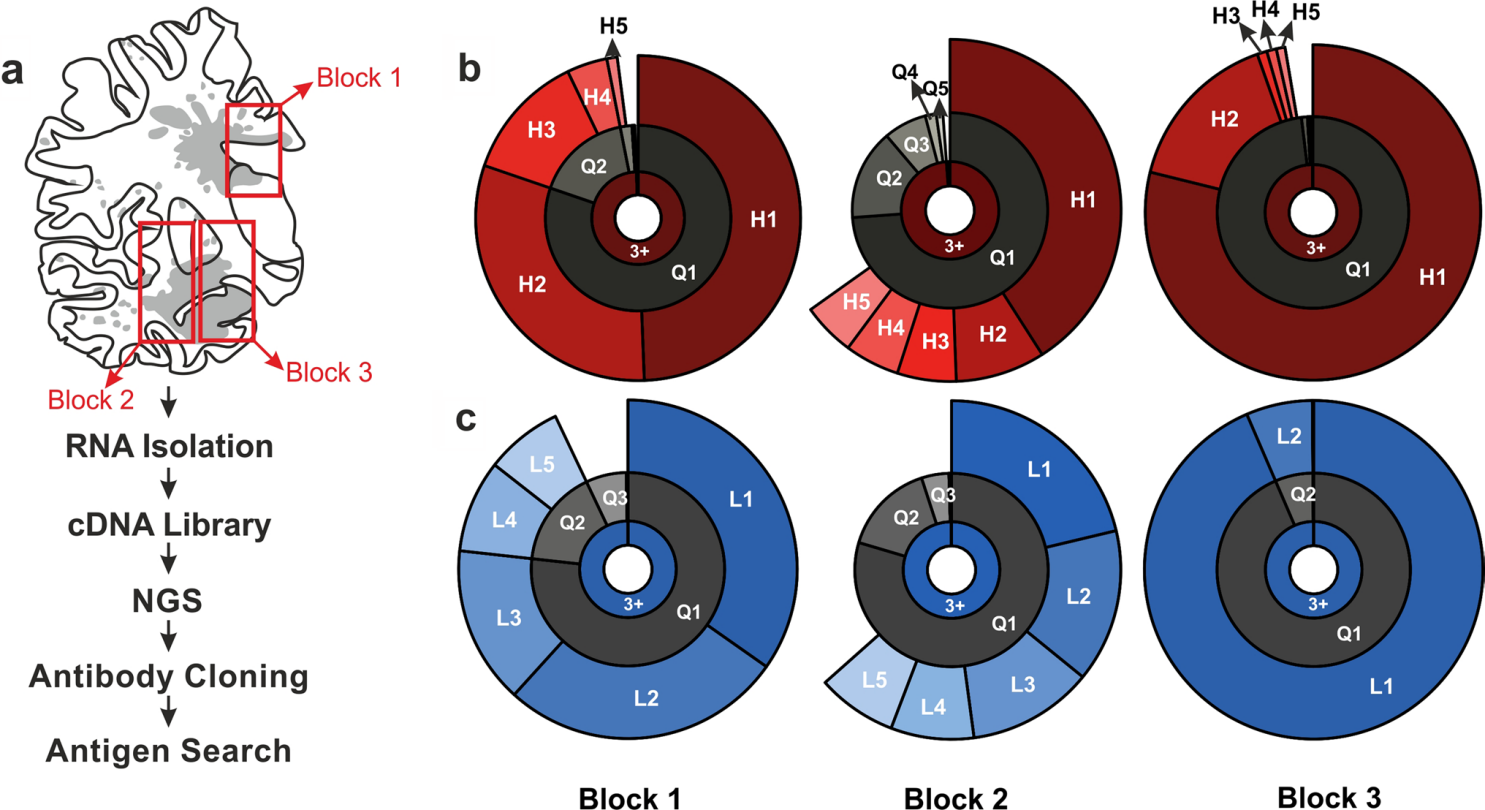
Clonal distribution of IgG H- and L-chains in three tissue blocks of patient hAE. **a** Camera lucida drawing of a large brain sections from the hAE patient with topographical distribution of demyelinated lesions (grey) and of the 3 tissue blocks used (red boxes). The flow chart summarizes the experimental procedures. Clonality plot of H-chain (H, **b**) and L-chain immunoglobulins (L, **c**) found in blocks 1 to 3. The result of the repertoire clonality analysis for each region and chain is plotted on a three-layer donut chart. The inner layer includes the frequency of unique nucleotide sequences that arise during the gene rearrangement process for individual chains (clonotypes) that were identified three or more times (“3+”). The middle layer (“quantile”) displays the abundance of top 20% (“Q1”), next 20% (“Q2”) and further 20% (Q3, Q4, Q5) clonotypes. The outer layer displays the individual abundance of top 5 clonotypes

To investigate the antibody repertoire expressed by B- and plasma cells in the lesions, we isolated RNA from three different regions of brain (Fig. 2a). The samples were deparaffinised, treated with citraconic anhydride to partly reverse formylation, and fragmented RNA was isolated by chromatography. Sufficient RNA quality for generation of NGS libraries was confirmed by electrophoresis on microfluidic chips.

**Fig. 3:**
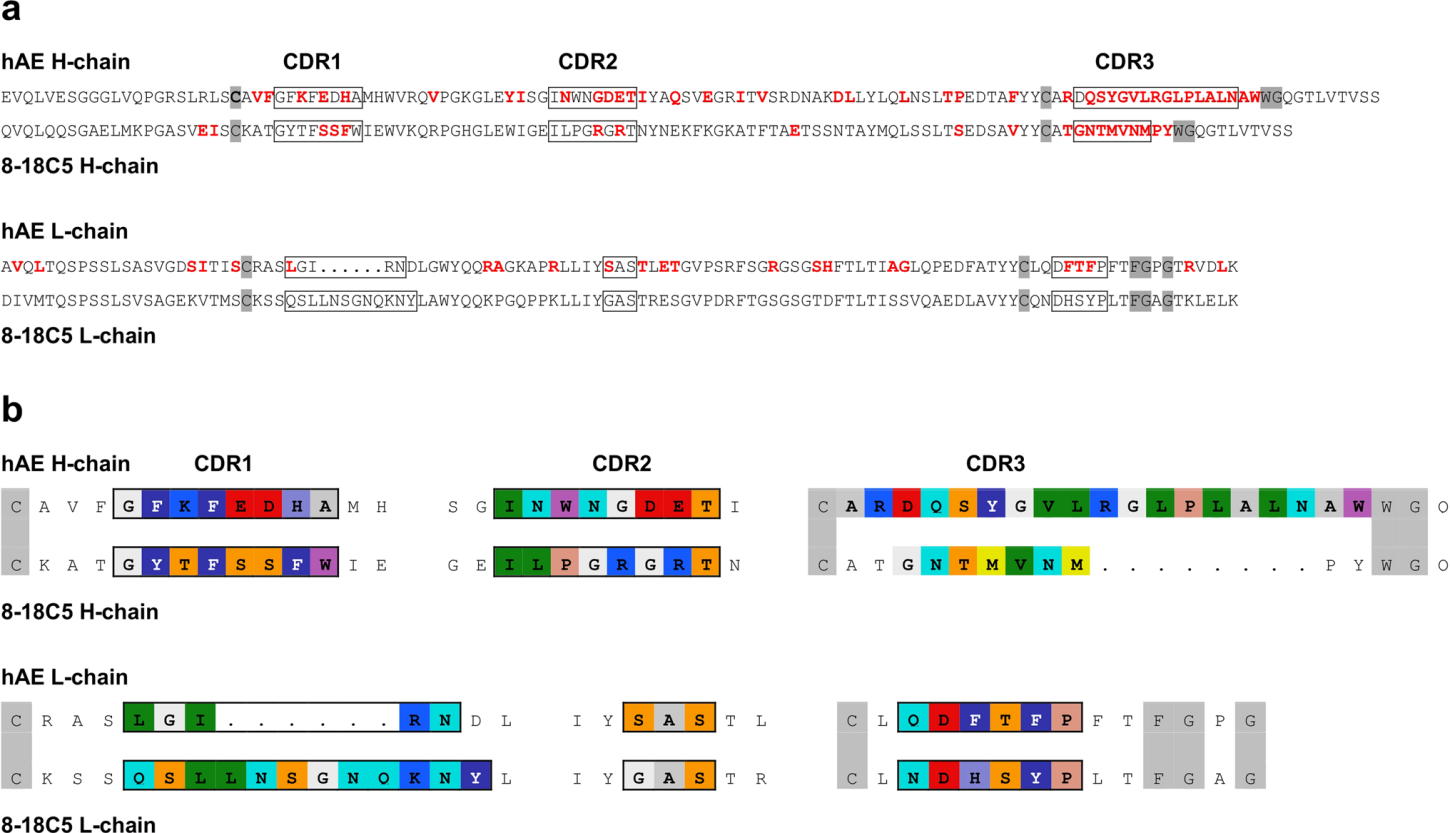
Comparison of the amino acid sequences of the H-and L-chains of Ab-hAE and 8-18C5. The sequences are shown in single letter code. Complementarity determining regions (CDR) were determined according to IMGT and are indicated in the uppermost line. **a** For clarity, amino acids of the CDRs are boxed. The conserved cysteine residues in the V-regions and the amino acids WG and FGxG in the J-regions of the H- and L-chains, respectively, which are structural guideposts, are highlighted in grey. To illustrate affinity maturation, amino acids that comprise the CDR3 regions or were introduced by SHM are shown in red letters. Germline-encoded amino acids are shown in black. The germline alleles are: 8-18C5 H-chain: mouse IGHV1-18*04, 8-18C5 L-chain: mouse IGKV8-28*01, rAb-hAE H-chain: human IGHV3-9*01, and rAb-hAE L-chain: human IGKV1-6*01. **b** To compare the amino acid composition of the loops that may contact antigens, the most prominent amino acids of the CDR regions are shown in colors. Color code is according to RasMol, e.g., red: negatively charged, blue: positively charged, light blue: big hydrophilic, dark blue with white letters: aromatic amino acids, green: aliphatic amino acids

To identify immunoglobulin chains by NGS we used two different cDNA libraries. First, we generated an antibody-specific library using primers specific for the H- and L-chains (Ig-seq). The primers were designed to hybridize to the 3′-end of the conserved regions and to allow for upstream reads. These libraries revealed the CDR3 regions of all H- and L-chains. Figure 2b, c shows the clonal distribution of the CDR3 regions of the most prominent H- and L-chains for the three lesions as cake diagrams. In block 3, we detected an almost monoclonal expression of one H- and one L-chain. These chains, designated H1 and L1, were also the dominant chains in blocks 1 and 2 although several other less expanded chains were observed in addition. This analysis revealed only the CDR3 regions of the antibody chains but not the CDR1 and CDR2 regions, which also may contribute to antigen recognition. To reconstruct the full-length sequences of the H1 and L1 chains, we generated a second library that contained the whole transcriptomes of the three blocks. From this library, we assembled in silico step by step the full-length sequences of both chains using the CDR3 sequences as start. The amino acid sequences of the H1 and L1 chains are shown in Fig. 3a. The CDR regions and amino acids introduced by somatic hypermutation (SHM) are indicated. Class switch to IgG and the presence of a high number of SHMs provides evidence for antigen driven maturation.

### The recombinant antibody recognizes myelin oligodendrocyte glycoprotein (MOG)

**Fig. 4:**
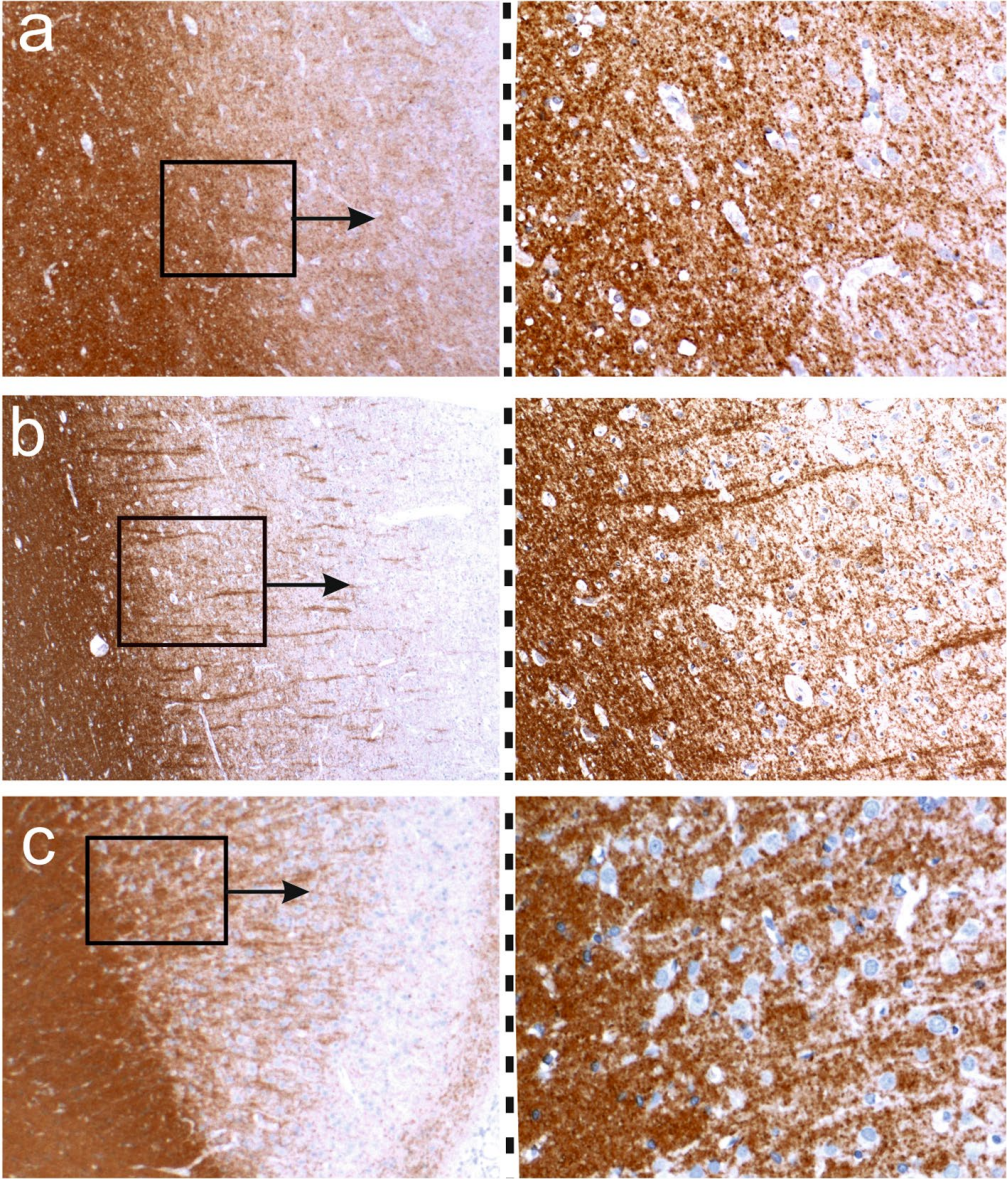
rAB-hAE reacts with myelin. Myelinated brain sections of the hAE patient (**a**), of a human control patient (**b**), and of a Lewis rat (**c**) were reacted with rAB-hAE. Positive reaction products are brown, and the tissue was counterstained with hematoxylin to show the nuclei in blue. The boxes with arrows mark details which are enlarged in the adjacent pictures

To produce a functional recombinant antibody, we synthesized the Vn(D)nJ-regions of both chains, inserted them into pTT5 expression vectors that contained the conserved regions of the IgG1 H- and kappa chains, and expressed the recombinant antibody rAb-hAE in HEK293-Expi293 cells. Staining of tissue sections of primate brain indicated that rAb-hAE recognizes myelin (Suppl. Fig. 1). Next, we performed immunohistochemistry on formaldehyde fixed and paraffin embedded brain tissue from Lewis rats, a healthy human control autopsy, and the hAE case. rAb-hAE showed strong and distinct myelin staining in the white matter and of myelinated axons in the cortex (Fig. 4). The binding intensity among these three tissue types (rodent tissue, human control tissue and hAE tissue) was similar and allowed a good discrimination of the white and gray matter within the CNS.

**Fig. 5:**
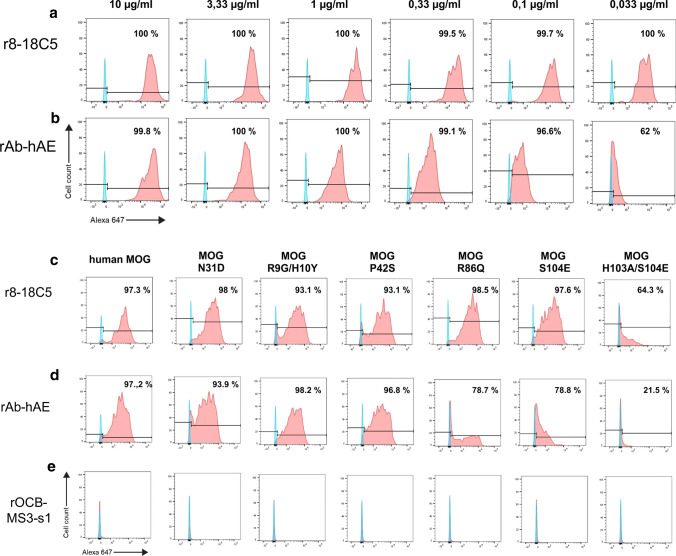
Recognition of hMOG. **a**, **b** COS-7 cells were transiently transfected with full length hMOG and analyzed by flow cytometry for hMOG-binding of rAb-hAE. The cells were stained with the control antibody r8-18C5 (**a**) and rAb-hAE (**b**) at concentrations ranging from 10 to 0.033 µg/ml. The percentages of positive cells are indicated in the plots. Both antibodies recognized hMOG but the reduced shift of rAb-hAE below 1 µg/ml indicated a lower affinity for hMOG by rAb-hAE as compared to r8-18-C5. To determine the epitope of rAB-hAE, COS-7 cells were transiently transfected with full length hMOG or hMOG-mutants and analyzed for recognition of r8-18C5 (**c**), rAb-hAE (**d**), and the negative control antibody rOCB-MS3-s1 (**e**) by flow cytometry. For each sample, 30 µg/ml antibody were used. The amino acid substitutions are indicated at the top and the percentages of positive cells are indicated in the plots. Substitutions of histidine 103 and serine 104 by alanine and glutamine acid showed a strong decrease of hMOG-binding for rAb-hAE, proving that these amino acids are the dominant part of the epitope. The single substitutions S104E and R86Q had minor effects only on rAb-hAE but not on r8-18C5

Having established its binding to myelin, we tested whether rAb-hAE might recognize MOG. To this end, we used COS-7 cells transfected with full length human MOG (hMOG [32]), and measured binding of rAb-hAE antibody and r8-18C5. Figure 5 shows that both antibodies, rAb-hAE and r8-18C5, recognize hMOG, whereas rOCB-MS3-s1 did not. Of note, the affinity of rAb-hAE to MOG was lower than of r8-18C5, because titration of r8-18C5 and rAb-hAE (Fig. 5a, b) showed that at concentrations lower than 3.33 µg/ml the shift of rAb-hAE was significantly diminished as compared to r8-18C5. To identify the epitope of rAb-hAE, we used COS-7 cells transfected with wild-type hMOG and with MOG variants that had mutated amino acids at prominent surface exposed loops of the extracellular domain [32]. We compared rAb-hAE and r8-18C5 and included antibody rOCB-MS3-s1 as negative control (Fig. 5c–e). Flow cytometry provided evidence that rAb-hAE recognizes the loop that contains histidine 103 and serine 104. Substitution of S104 by glutamic acid (S104E) had a minor, though significant effect on rAb-hAE but had almost no effect on r8-18C5 (Fig. 5d, e, second to right column). Concomitant substitution of S104 and of H103 by alanine (H103A) abrogated binding of rAb-hAE almost completely, whereas binding of r8-18C5 was only diminished in the double-mutant H103A/S104E (Fig. 5d, e, rightmost columns). The slight differences between rAb-hAE and r8-18C5 may be due to minor differences in the epitopes or to the reduced affinity of rAb-hAE. All other substitutions did not affect recognition of MOG by both, rAb-hAE and r8-18C5. A titration of rAb-hAE (Suppl. Figure 2) showed dose-dependency of recognition of the double mutant H103A/S104E and the single mutant S104E as compared to the wild type MOG and the tolerated exchange R86Q. This illustrates the relevance of this loop as the major epitope of rAb-hAE. Furthermore, the recognition of rat and mouse MOG was identical to that of human MOG (Suppl. Fig. 3) despite 13 and 12 amino acid exchanges in the extracellular domains, respectively. Of note, amino acids H103 and S104 are conserved in humans and rodents supporting our finding that rAb-hAE recognizes the same epitope as r8-18C5.

### Different amino acid sequences of r8-18C5 and rAb-hAE recognize highly similar epitopes of hMOG

As the antibodies r8-18C5 and rAb-hAE show a very similar binding pattern, we compared the amino acid sequences of these antibodies (Fig. 3b). The H-chains had different charges in the CDR1 (one positive and two negative charges vs. all neutral amino acids), and CDR2 (two negative charges vs. two positive charges), and striking differences in charges, hydrophobicity and length in the CDR3 regions. When comparing the L-chains, we found in the CDR1 one conserved positive charge but very different lengths. The CDR2 and CDR3 regions are almost identical considering that Histidine (H in CDR3 of the 8-18C5 L-chain) is aromatic at neutral and alkaline pH and thus homologous to the corresponding F in the rAb L-chain. Thus, all three CDRs of the H-chains and the CDR1 of the L-chains of Ab-hAE and 8-18C5 are very different but CDR2 and CDR3 of the L-chains are nearly identical. This is remarkable as it is known that it is the H-chain of r8-18C5 that is preferentially interacting with the loop containing H103 and S104 of MOG [7], but a structural correspondence of the H-chains of 8-18C5 and Ab-hAE is missing.

### rAb-hAE antibody shows in vivo demyelinating activity

**Fig. 6:**
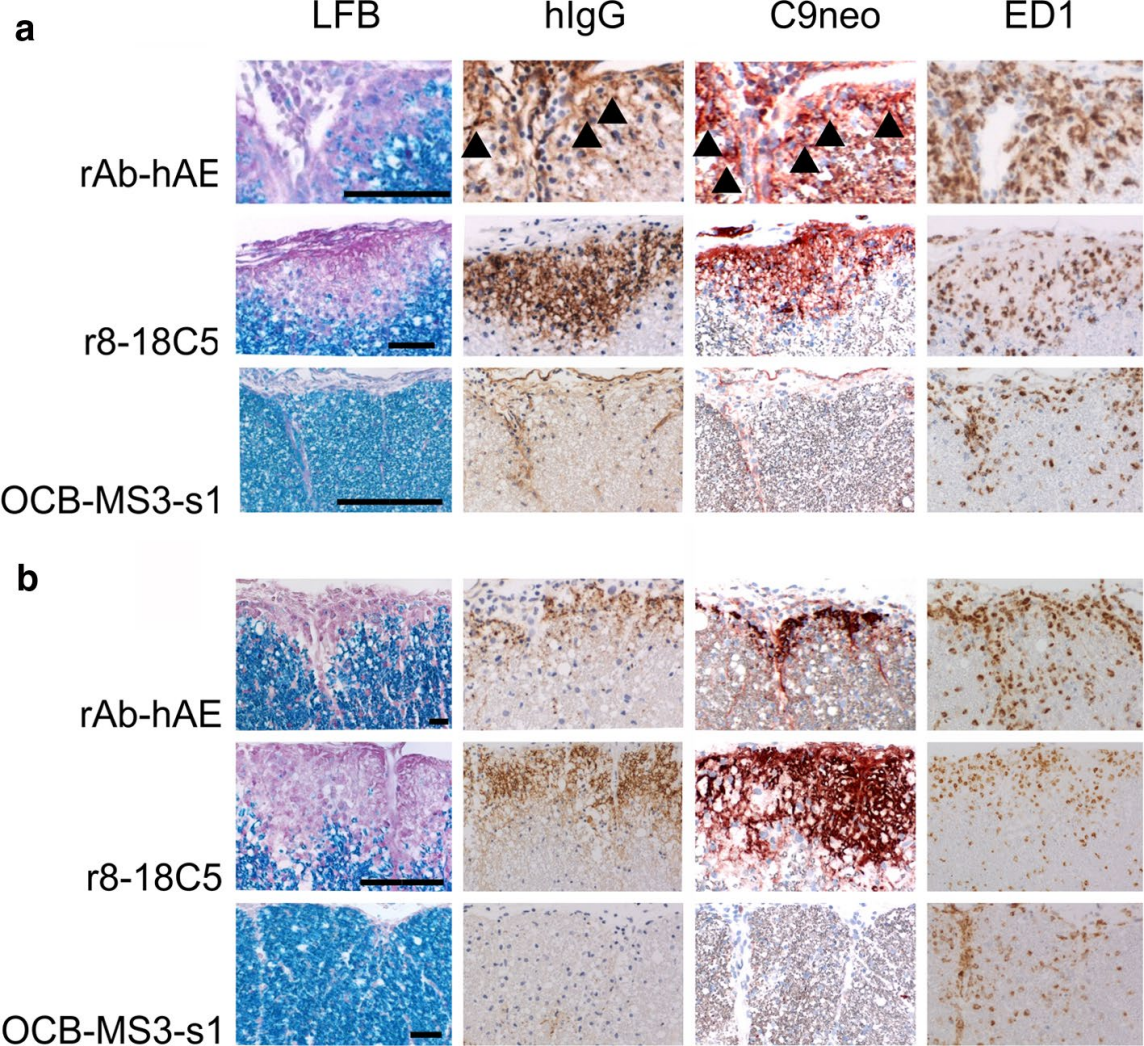
Demyelinating activity of rAB-hAE in vivo. Spinal cord sections of Lewis rats with MBP-specific T-cell induced experimental autoimmune encephalomyelitis, which have been intraperitoneally (**a**) or intrathecally (**b**) injected with rAB-hAE, r8-18C5 (positive control) or OCB-MS3-s1 (negative control). The tissues were stained with Luxol Fast Blue (LFB), antibodies against human IgG (brown, in case of weaker staining also arrowheads), against complement C9neo (red, in case of weaker staining also arrowheads), or with the ED1 antibody (brown). Bars 50 µm (first lane) or 100 µm (all other lanes)

To proof the pathogenicity of the rAb-hAE antibody we conducted two EAE experiments with Lewis rats. For that, we firstly injected activated myelin basic protein (MBP)-specific T cells (i.p.) on day 0, followed by an i.p. injection of the recombinant antibodies rAb-hAE, r8-18C5 or rOCB-MS3-s1 on day 4, when the animals had an average clinical score of 1 (Table 6). On day 6, when the animals were sacrificed, all rOCB-MS3-s1 injected animals had a clinical score of 1.5, while the r8-18C5- and rAB-hAE-injected animals had average clinical scores of 2.9 and 2, respectively (Table 6). The animals were sacrificed on day 6. In pathology, no significant differences were seen in the degree of inflammation between animals, injected with T-cells alone, with T-cells and the control antibody, the positive control antibody r8-18C5 or the rAb-hAE antibody (Fig. 6a). However, differences were evident in the extent of demyelination. R8-18C5 used as positive control caused profound subpial demyelination within the spinal cord, associated with the deposition of immunoglobulins and complement C9neo on myelin sheaths, and locally increased numbers of ED1^+^ macrophages/activated microglia cells in the areas of myelin damage. Demyelination was present in the rAb-hAE antibody-treated animals as well, but it was less extensive than that seen in the r8-18C5-injected animals. However, also in the rAb-hAE antibody-treated animals, demyelination of the subpial spinal cord occurred in the presence of human immunoglobulin and complement C9 neo deposition, associated with increased numbers of ED1^+^ macrophages/activated microglial cells (Fig. 6a). There was no demyelination in the animals injected with the negative control antibody rOCB-MS3-s1.

**Table 6:** Clinical scores of animals with EAE, injected intraperitoneally with different rABs

	Day 4	Day 6
1 mg r8-18C5 (*n* = 4)	1; 0.5; 1.5; 1	2.5; 3; 3; 3
1 mg rOCB-MS3-s1 (*n* = 3)	1; 1.5; 0.5	1.5; 1.5; 1.5
4 mg rAB-hAE (*n* = 4)	1; 1.5; 0.5; 1	2.5; 2; 2; 1.5
4 mg rOCB-MS3-s1 (*n* = 2)	0.5; 1	1.5; 1.5

We then reproduced these experiments using a different transfer system, which has been used to identify pathogenic human anti-MOG antibodies from patients with MOGAD [43]. In this experimental set up, brain inflammation was induced by systemic transfer of MBP-reactive T-cell lines, but the respective antibodies were directly injected into the cerebrospinal fluid via the cisterna magna. The outcome of this experiment was the same as described before with systemic antibody injection (Fig. 6b). We did not find an effect on the degree of inflammation, but subpial demyelination was induced by the positive control antibody r8-18C5 and the rAB-hAE antibody, but absent in animals injected with the control antibody rOCB-MS3-s1. In this experiment, too, demyelination was associated with subpial deposition of human immunoglobulin and activated complement (C9neo antigen).

## Discussion

Our study shows that it is possible to identify the target antigen of an autoimmune response from an archival formalin fixed and paraffin embedded brain tissue block of a patient, who died more than 60 years ago. This offered the unique chance to define the pathogenesis of an autoimmune disease, which gave rise to brain lesions with a degree of similarity to multiple sclerosis, which so far has never been achieved in experimental autoimmune encephalomyelitis in rodents or primates [13].

Identification and resurrection of the pathogenic antibody became possible for mainly two reasons: First, in block 4 of the autopsy sample, we detected an almost monoclonal expansion of one antibody species as seen by dominant expression of one H- and one L-chain. This allowed us to unambiguously relate matching H- and L-chains. In blocks 2 and 3, these chains were also expressed at high levels suggesting that one plasma cell clone was pervasive in the entire brain. Second, after having identified the expanded chains by Ig-seq, we used these data and combined them with data obtained from an RNA-seq library that contained about 10^6^ short fragments covering the whole transcriptome. Thus, it was possible to assemble in silico the full-length sequences, although NGS reads are particularly short when FFPE fixed tissue is used, because frequent formylation of nucleotides may terminate strand extension during reverse transcription and PCR and CCA treatment may only partially reverse formylation.

The amino acid sequences of the Ab-hAE chains contain many exchanges from the germline sequence, in particular in CDR1 and CDR2, which both may contribute to antigen binding. This shows that the pathogenic antibody has undergone extensive SHM, which requires sustained exposure to antigen and help by CD4^+^ T cells, although we did not see any evidence for CD4^+^ T-cell / B-cell interaction within the brain lesions. Comparison of the CDR sequences to the amino acid sequences of 8-18C5 chains reveals that all CDRs are very different, except CDR2 and CDR3 of the L-chains. Of note, the MOG amino acids H103 and S104, which protrude from the surface of MOG [7] are the dominant amino acids in the epitopes of both antibodies, although at least 8-18C5 binds these amino acids with the H-chains only. Thus, highly similar antigenic structures were recognized by structurally very different antibody loops, which underlines that not only T cell receptors but also antibodies may be highly polyspecific [50].

The epitope containing H104 and S104 recognized by rAb-hAE and r8-18C5 is located at the membrane-distal part of the extracellular domain of MOG, and is probably therefore more immunogenic than other regions. Strong antibody responses against this immunodominant epitope were also seen when mice were immunized with MOG DNA in an expression plasmid [6]. In the patient with hAE, sustained stimulation by antigen was granted by seven immunizations during 17 months. In this preparation, which contained lyophilized calf brain but no CFA, MOG molecules amounted to almost negligible quantities, but still initiated a vigorous immune response to MOG. Antibody 8-18C5, by contrast, was obtained after immunization with rat myelin glycoproteins in presence of CFA [27]. It is remarkable that both antibodies were generated by immunization with crude antigen-preparations and recognize closely related, if not identical, epitopes on the FG-loop on MOG, despite the fact that the immunogens came from different animal species. This is in contrast to the variety of epitopes on the extracellular surface of MOG that were recognized by antibodies from patients with MOG-antibody associated disease (MOGAD) who were not actively immunized [32, 43, 47]. The reason for this difference is unknown.

The models of experimental autoimmune encephalomyelitis in rodents and primates, which show the most extensive primary demyelination, are driven by T-cell mediated brain inflammation, which is amplified by the presence of a demyelinating antibody response [26, 28]. This is achieved by sensitization with crude preparation of CNS tissue or myelin as well as by sensitization with full length recombinant MOG [25]. Although immunization with CNS tissue gives rise to many different autoantibodies, demyelinating auto-antibodies have a surprisingly restricted recognition repertoire, most of them being directed against conformational epitopes of myelin oligodendrocyte glycoprotein [4, 6]. This was the reason, why we tested the resurrected antibody from this case against MOG and not against other myelin proteins. Since the pathogenic epitope, recognized by MOG antibodies is exclusively expressed on myelin and oligodendrocytes, MOG antibody associated lesions in the central nervous system show primary demyelination, while axons are largely preserved [11, 17]. Our study for the first time provides evidence that also humans, when immunized with brain tissue, mount an antibody response against a conformational epitope of MOG. Our data further support the view that this antibody response was pathogenic, since it was associated with inflammatory demyelination in the original patient, the demyelination was associated with antibody deposition and complement activation at sites of active demyelination [13] and our reconstructed recombinant antibody induced complement mediated demyelination in vivo after transfer into rats with inflammatory brain disease.

The presence of auto-antibodies against conformational epitopes of MOG has recently been shown in a subset of patients with inflammatory demyelinating disease [34, 37, 38] and it also was shown that these antibodies can induce demyelination in vitro and in vivo [35, 43]. Although these diseases share clinical, pathological and immunological features of multiple sclerosis, it is increasingly suggested that MOGAD is an entity distinct from MS with regard to clinical course [17, 18, 20, 38] and pathology [14, 46]. MOGAD is an acute monophasic or relapsing inflammatory demyelinating disease. However, these antibodies, although also present in patients with long standing chronic relapsing disease, are typically not found in patients with secondary or primary progressive MS and similarly, no transition from relapsing to progressive disease has been reported so far in MOGAD patients.

Thus, the presence of anti-MOG antibodies in our case of human autoimmune encephalomyelitis suggests that the disease process has to be classified as MOGAD. Interestingly, however, pathology in the particular case described here, showed much more similarities to MS [13], compared to that in spontaneous MOG antibody associated disease [14]. Although MOGAD and MS both are inflammatory demyelinating diseases, they are distinct in several important aspects [14, 46]. The inflammatory reaction in MOGAD is dominated by CD4^+^ T-lymphocytes with only sparse CD8^+^ cells and CD20^+^ B-cells. The opposite is seen in multiple sclerosis, where the inflammatory infiltrates are mainly composed of CD8^+^ T-cells and CD20^+^ B-cells, while CD4^+^ T-cells are present only in very low numbers [10, 29, 49]. Demyelination in MOGAD is mainly perivenous around small to middle sized veins and venules with confluence of adjacent perivenous sleeves of demyelination. In contrast, demyelination in MS occurs mainly around larger (periventricular) veins with radial expansion of the lesions and perivenous expansion of the lesions (Dawson fingers [12]). In cortical lesions small perivenous intracortical lesions dominate in MOGAD, while the majority of cortical lesions in MS are subpial lesions, related to meningeal inflammatory infiltrates [22, 30]. A key difference between these two diseases is related to the chronic expansion of pre-existing lesions, which is prominent in MS patients and related to disability progression [8], but absent in MOGAD [14, 46]. Interestingly, the case of hAE, described here, reproduced the pathological spectrum of MS and not that of MOGAD [13], despite the presence of anti-MOG antibodies.

The properties of autoantibodies against MOG in human inflammatory demyelinating disease of the CNS have been characterized in several recent studies [32, 47]. Therefore, we compared the binding pattern of the recombinant rAb-hAE antibody with anti-MOG antibodies described by before. Mayer et al. [32] isolated anti-MOG antibodies from a patient cohort suffering from patients with different disease phenotypes, like acute disseminated encephalomyelitis (ADEM), transverse myelitis or optic neuritis, multiple sclerosis (MS), anti-aquaporin-4 (AQP4)-negative neuromyelitis optica (NMO) and chronic relapsing inflammatory optic neuritis (CRION) and compared the antibodies’ binding pattern to different MOG mutants. When comparing the binding pattern of the rAb-hAE antibody with the heterogeneous anti-MOG antibodies from this patient cohort, we observed a binding pattern similar to an anti-MOG antibody isolated from an ADEM patient, which has been reproduced in a larger series of MOGAD patients [47]. This finding indicates that the rAb-hAE antibody identified from this unique hAE case might be within the heterogeneous antibody spectrum found in patients suffering from different MOG antibody associated CNS autoimmune diseases, and it is, therefore, unlikely, that the differences in histopathology between MOGAD and the hAE case is explained by the properties of the pathogenic antibody. The pairing of the further identified immunoglobulin chains of this unique case and the antigen recognition of these antibodies remains elusive, but could be the result of epitope spreading due to tissue damage induced by the initial immune reaction.

### Limitations of the present study

The most important limitation of our study is that the identification of the target antigen of the immune response is based on observations in a single case and there is currently no possibility to change this situation. In the entire medical literature only 8 other patients have been described, who developed an MS like inflammatory demyelinating disease, similar to that observed in our study [48]. We have tried to retrieve tissue from the other cases with-MS like pathology from the neuropathological archives, but were told that the respective material was no longer existing. All other brain tissue immunized patients were affected clinically and/or pathologically by Guillain Barre Syndrome or acute disseminated encephalomyelitis (ADEM; [23, 45]). It is currently unresolved, to what extent the ADEM cases show similarities in their inflammatory reaction and demyelination to MOGAD, and whether they have an immune response against MOG, which is different from this particular case. Finally, although the dominant antibody response in the hAE case is directed against MOG, we cannot formally exclude the existence of additional antibodies directed against other autoantigens of the CNS.

## Conclusions

Historically, the key argument for MS being an autoimmune disease came from the pathological description of a very small number of cases in the 1950^ths^, who developed a disease closely resembling MS after immunization with brain tissue [40, 48]. In our present study we show that autoimmunity in one of these cases is directed against MOG, and that the diagnosis in this case is MOGAD and not multiple sclerosis.

## Electronic supplementary material

Below is the link to the electronic supplementary material.

Supplementary file1 Suppl. Figure 1: Immunohistochemistry reveals identical binding pattern of rAb-hAE and r8-18C5 to primate cerebellum. Commercial slices of primate cerebellum (Euroimmun, Lübeck, Germany) were stained with rAb-hAE (a), the anti-MOG antibody r8-18C5 (b), and the negative control antibody rOCB-NB1-s13 (c). All antibodies were detected using biotinylated mouse anti-human IgG1 and a Streptavidin Alexa FluorTM488 conjugate (green). Cell nuclei are labelled using DAPI staining (right column). Bar = 100 µm (JPG 1913 kb)

Supplementary file2 Suppl. Figure 2: Dose-dependent recognition of hMOG variants by rAb-hAE. Flow cytometry of transiently transfected COS-7 with hMOG (upper panel) and the three mutants R86Q (second panel), S104E (third panel), and H103A/S104E (double-mutant, lowest panel) showed the dose-dependency of the rAb-hAE binding. The concentrations of rAb-8-18C5 ranged from 100 µg/ml to 0,033 µg/ml. The percentages of positive cells are indicated in the plots. The double mutant H103A/S104E had the strongest effect on binding of rAb-hAE followed by S104E. Mutant R86Q showed about the same binding pattern as wild type hMOG (JPG 688 kb)

Supplementary file3 Suppl. Figure 3: Recognition of MOG from different species by rAb-hAE, r8-18C5, and rOCB-MS3-s1. COS-7 cells were transiently transfected with MOG variants and analyzed by flow cytometry. rAb-hAE and r8-18C5 recognized hMOG, mouse MOG (mMOG) and rat MOG (rMOG) (JPG 434 kb)
