# Supplementary figures and images for: Archeological neuroimmunology: resurrection of a pathogenic immune response from a historical case sheds light on human autoimmune encephalomyelitis and multiple sclerosis
Source: Acta Neuropathol. 2020 Oct 29;141(1):67–83. doi: 10.1007/s00401-020-02239-2 (PMC7785560; doi:10.1007/s00401-020-02239-2)

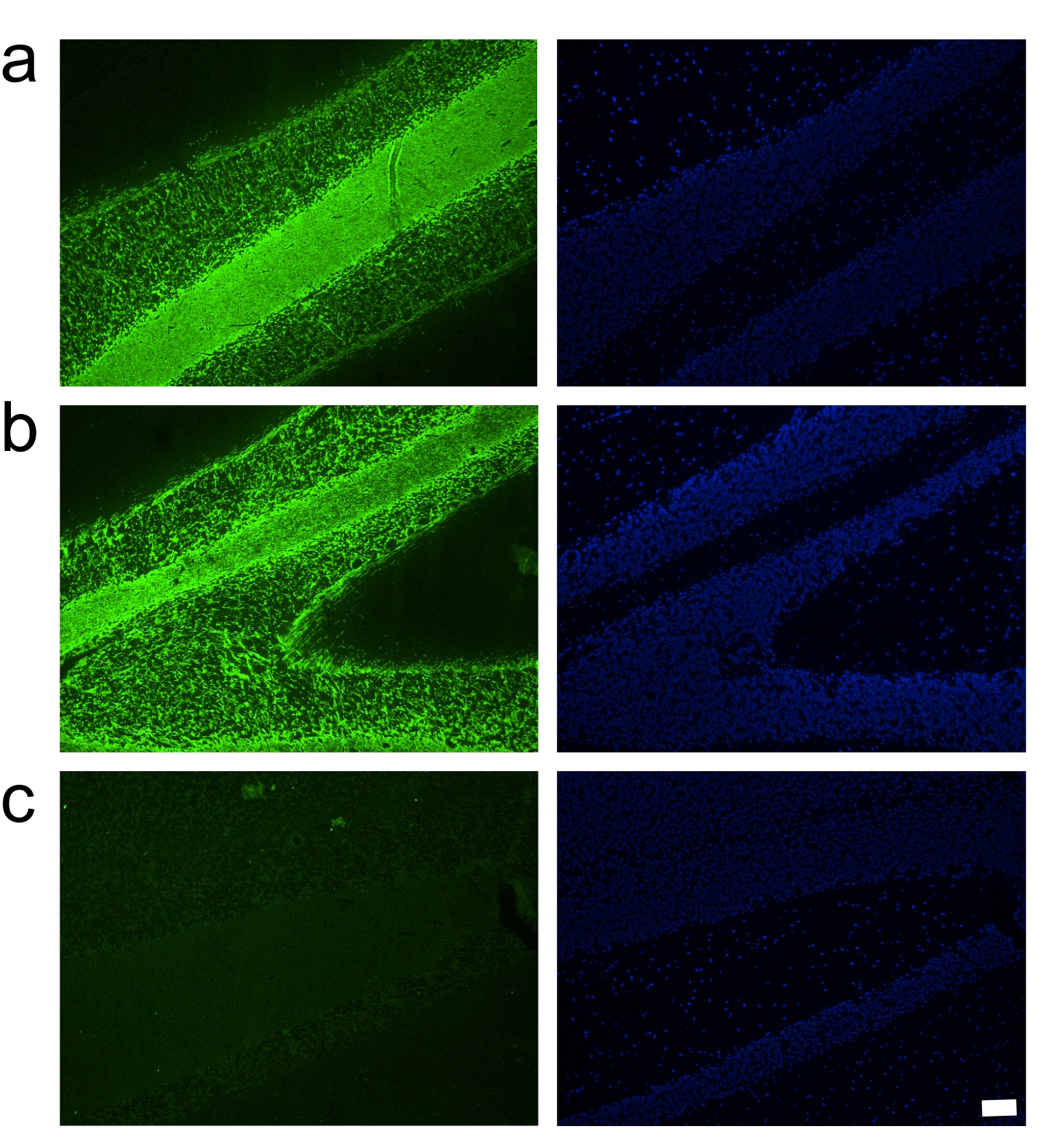

Supplement: Supplementary file 1 — Supplementary file1 Suppl. Figure 1: Immunohistochemistry reveals identical binding pattern of rAb-hAE and r8-18C5 to primate cerebellum. Commercial slices of primate cerebellum (Euroimmun, Lübeck, Germany) were stained with rAb-hAE (a), the anti-MOG antibody r8-18C5 (b), and the negative control antibody rOCB-NB1-s13 (c). All antibodies were detected using biotinylated mouse anti-human IgG1 and a Streptavidin Alexa FluorTM488 conjugate (green). Cell nuclei are labelled using DAPI staining (right column). Bar = 100 µm (JPG 1913 kb) [file 401_2020_2239_MOESM1_ESM.jpg]

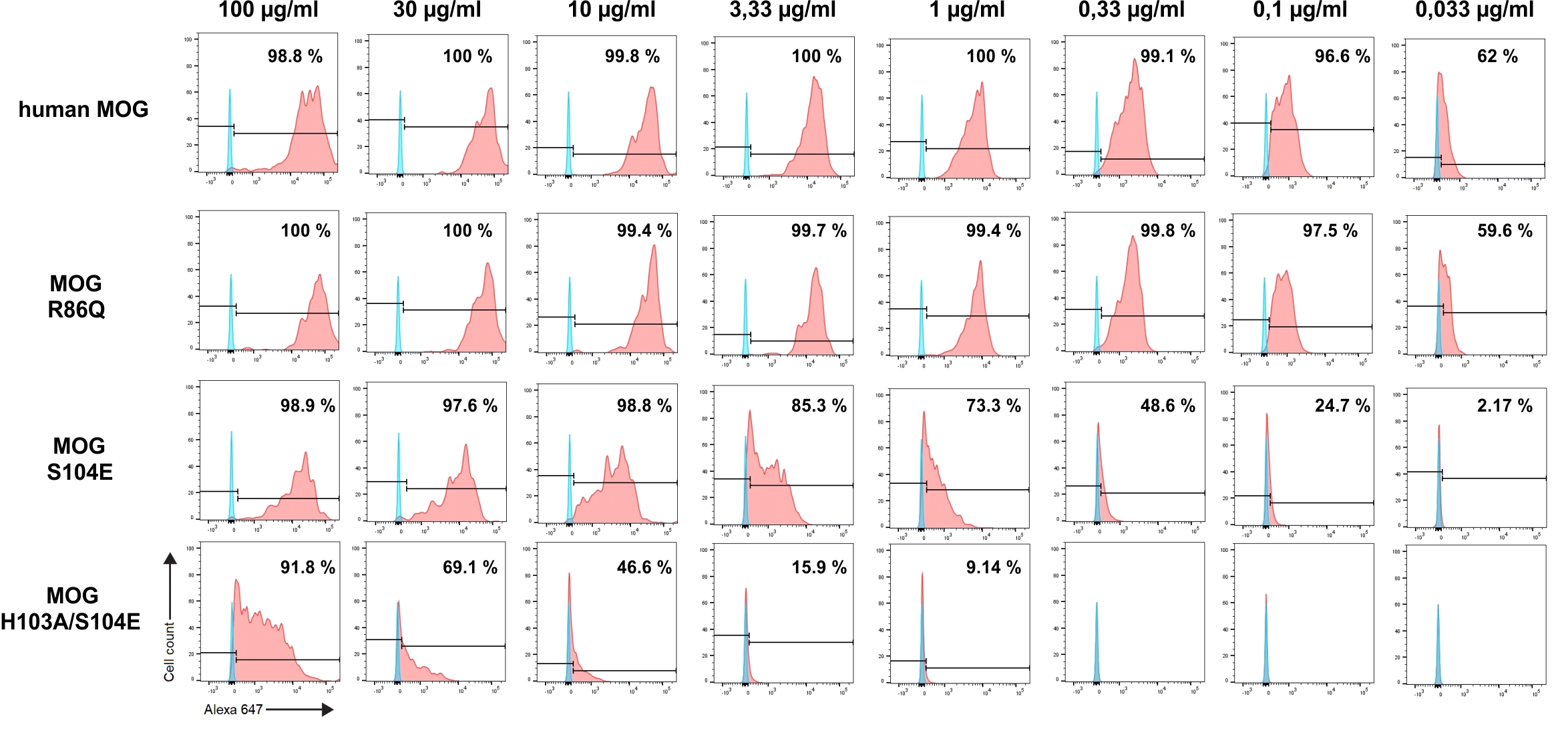

Supplement: Supplementary file 2 — Supplementary file2 Suppl. Figure 2: Dose-dependent recognition of hMOG variants by rAb-hAE. Flow cytometry of transiently transfected COS-7 with hMOG (upper panel) and the three mutants R86Q (second panel), S104E (third panel), and H103A/S104E (double-mutant, lowest panel) showed the dose-dependency of the rAb-hAE binding. The concentrations of rAb-8-18C5 ranged from 100 µg/ml to 0,033 µg/ml. The percentages of positive cells are indicated in the plots. The double mutant H103A/S104E had the strongest effect on binding of rAb-hAE followed by S104E. Mutant R86Q showed about the same binding pattern as wild type hMOG (JPG 688 kb) [file 401_2020_2239_MOESM2_ESM.jpg]

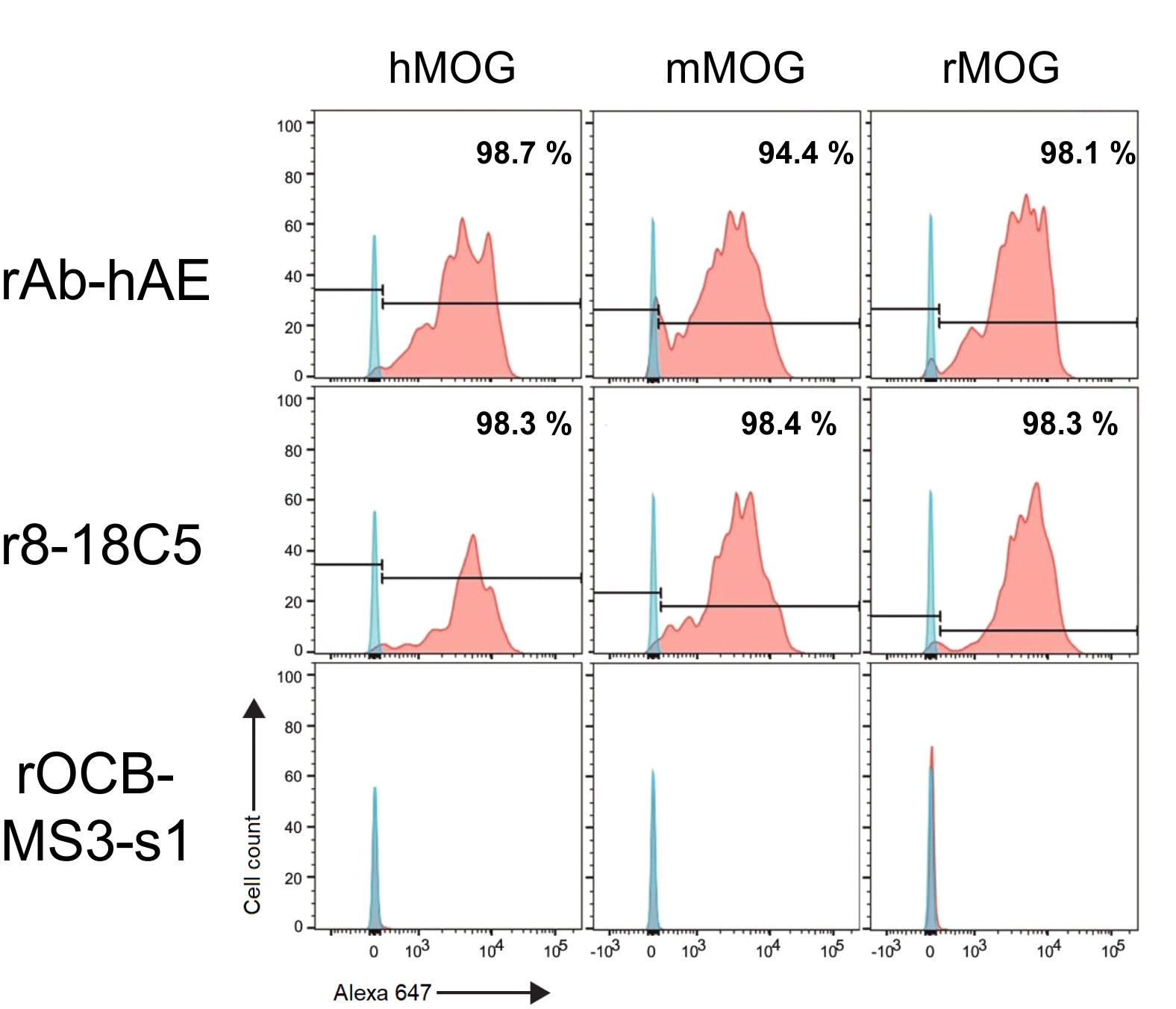

Supplement: Supplementary file 3 — Supplementary file3 Suppl. Figure 3: Recognition of MOG from different species by rAb-hAE, r8-18C5, and rOCB-MS3-s1. COS-7 cells were transiently transfected with MOG variants and analyzed by flow cytometry. rAb-hAE and r8-18C5 recognized hMOG, mouse MOG (mMOG) and rat MOG (rMOG) (JPG 434 kb) [file 401_2020_2239_MOESM3_ESM.jpg]
